# Reliable assessment of carbon black nanomaterial of a variety of cell culture media for in vitro toxicity assays by asymmetrical flow field-flow fractionation

**DOI:** 10.1007/s00216-023-04597-8

**Published:** 2023-02-25

**Authors:** Aaron Boughbina-Portolés, Lorenzo Sanjuan-Navarro, Lusine Hakobyan, Marta Gómez-Ferrer, Yolanda Moliner-Martínez, Pilar Sepúlveda, Pilar Campíns-Falcó

**Affiliations:** 1 Departament de Química Analítica, Facultat de Química, Universitat de València, Dr. Moliner 50, 46100 Burjassot, Valencia Spain; 2 Regenerative Medicine and Heart Transplantation Unit, Instituto de Investigación Sanitaria La Fe, Avda. Fernando Abril Martorell 106, 46026 Valencia, Spain

**Keywords:** Carbon black, Cell culture media, Amino acid, Assessment, Toxicity

## Abstract

Carbon black nanomaterial (CB-NM), as an industrial product with a large number of applications, poses a high risk of exposure, and its impact on health needs to be assessed. The most common testing platform for engineered (E)NMs is in vitro toxicity assessment, which requires prior ENM dispersion, stabilization, and characterization in cell culture media. Here, asymmetric flow field-flow fractionation (AF4) coupled to UV–Vis and dynamic light scattering (DLS) detectors in series was used for the study of CB dispersions in cell culture media, optimizing instrumental variables and working conditions. It was possible to disperse CB in a non-ionic surfactant aqueous solution due to the steric effect provided by surfactant molecules attached on the CB surface which prevented agglomeration. The protection provided by the surfactant or by culture media alone was insufficient to ensure good dispersion stability needed for carrying out in vitro toxicity studies. On the other hand, cell culture media in combination with the surfactant improved dispersion stability considerably, enabling the generation of shorter particles and a more favourable zeta potential magnitude, leading to greater stability due to electrostatic repulsion. It was demonstrated that the presence of amino acids in the culture media improved the monodisperse nature and stability of the CB dispersions, and resulted in a turn towards more negative zeta potential values when the pH was above the amino acid isoelectric point (IEP). Culture media used in real cell culture scenarios were also tested, and in vitro toxicity assays were developed optimizing the compatible amount of surfactant.

**Graphical abstract:**

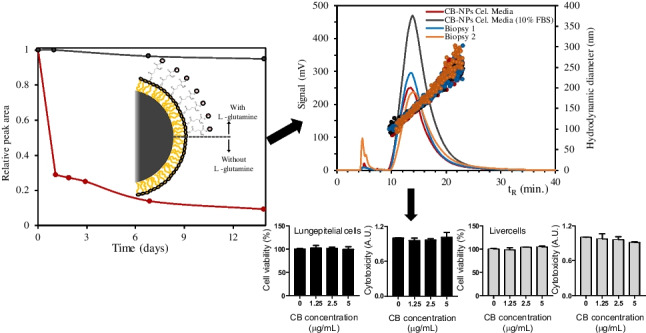

**Supplementary Information:**

The online version contains supplementary material available at 10.1007/s00216-023-04597-8.

## Introduction

Carbon black (CB) is a colloidal particulate material composed almost entirely of elemental carbon that looks like fine black granules or powder and is used in a variety of application areas such as pigments in inks and paints, tyre and plastic reinforcement, and batteries, due to its high electrical conductivity, staining capacity, weather resistance, and mechanical properties. Also, CB is used in a wide variety of key items, such as food contact grades, high-performance coatings, rubber goods, pipes, agricultural products, automotive components, and wires and cables [1, 2].

**Fig. 1:**
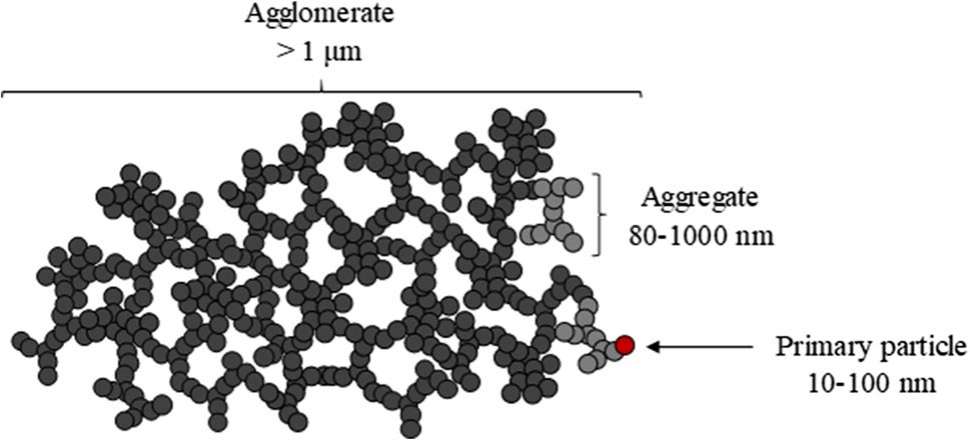
Schematic diagram of CB primary particles, aggregates, and agglomerates

CB nanopowders are produced industrially by incomplete combustion or thermal decomposition of solid, liquid, or gaseous hydrocarbons in a high-temperature-controlled environment. Structurally, CB is formed by primary particles or cores, also known as nodules, which can be between 10 and 100 nm in size. During their production, these nodules combine to form aggregates due to strong covalent bonding, which can range within approximately 80–1000 nm and consist of a few to several hundreds of nodules. These aggregates, which are considered to be the smallest dispersible unit of CB and can exhibit a wide variety of shapes, lead to the formation of larger unwanted agglomerates via van der Waals forces in highly polar environments like aqueous dispersions due to the high surface hydrophobicity of CB, as shown in Fig. 1 [2–4].

Advances in the knowledge, uses, and applications of CB inevitably lead to the exposure of the environment and humans to this nanomaterial (NM). Therefore, an understanding of its potential chronic effects is critical to minimizing hazardous effects to humans. Nanotoxicity not only depends on exposure route, dose, concentration time, and frequency, but also strongly varies as a function of physicochemical properties such as nanoparticle (NP) composition, shape, size, aggregation state, and surface chemistry. Obviously, the mechanism of interaction with biological systems such as organs, tissues, cells, and biomolecules will define the adverse effects of CB, and hence its physicochemical properties are a key factor for understanding these effects [5].

In vitro toxicity assessment of engineered NMs (ENMs), the most common testing platform for ENMs, generally requires prior ENM dispersion, stabilization, and characterization in cell culture media. Accurate assessment of the important properties of such polydisperse distributions including size distribution, effective density, charge, mobility, and aggregation kinetics, among others, is critical for understanding differences in the effective dose delivered to cells as a function of time and dispersion conditions, as well as for nano–bio interactions [6]. Afterwards, the tests measure a specific cellular endpoint likely to be altered by CB such as cell membrane morphology, cell apoptosis, cell death, genotoxicity, mitochondrial damage, reactive oxygen species (ROS) generation, and inflammatory and immune responses [7]. Recent reports revealed that an increase in ROS production is one of the most common alterations when cell lines are exposed to different pristine or functionalized CB, and hence is the main factor in CB toxicity [8, 9]. However, there are still controversial results and discussion regarding the real toxicity of NPs, and in particular of CB. Further studies are needed to evaluate the real impact on the environment and human health. Reliable in vitro toxicity results will be possible only if CB dispersion assessment is accurately and precisely performed. Hence, analytical nanotechnology must be able to offer standardized analytical methodologies that ensure the reliability of the subsequent in vitro toxicological data.

The size, shape, and aggregation of the CB can affect its cytotoxicity, cell uptake kinetics and interaction, and ultimately cell apoptosis. Surface chemistry, in particular surface charge, defines the interactions between CB and CB-biological system; thus, cellular uptake and kinetic transport will be governed by these interactions. It should be noted that phenomena such as agglomeration and aggregation have to be considered, since CB size—and hence the cellular uptake and kinetics—can vary. And in this context, stable dispersions in cell cultures that do not alter toxicological measurements are necessary [10–12].

NPs dispersed in biological fluids are easily coated by different types of biomolecules, such as carbohydrates, lipids, amino acids, or proteins [13, 14]. Hence, these biomolecules become part of the NP capping, the so-called NP corona, which can critically affect the interaction between NPs and their behaviour in complex matrices [14–16].

In recent years, various analytical techniques have been proposed for the study and characterization of CB-NP dispersions [2]. Fourier-transform infrared spectroscopy (FTIR), Raman spectroscopy, dynamic light scattering (DLS), and electron microscopy have been used to determine the physical and morphological properties of the CB-NPs. Electron microscopy was used here for studying the morphology of solid CB and CB dispersions. DLS works with dispersions and provides information about CB aggregate size and whether the dispersion is mono- or multimodal, but gives global information. This paper used DLS for estimating the zeta potential of several dispersions. Nevertheless, none of the mentioned techniques gives particle size characterization simultaneously with size-dependent separation and quantification. Asymmetrical flow field-flow fractionation (AF4) is a separation technique which provides information that allows us to evaluate the presence or absence of several NP distributions according to particle properties such as size, composition, or electrophoretic mobility [17–19]. AF4 covers a great size range, from a few nanometres to millimetres. Separation is controlled by cross-flow (hydraulic pressure gradient field) perpendicular to the channel. This flow drags the sample particles to the accumulation wall and gives rise to a concentration gradient, so that a size-dependent diffusion phenomenon towards the channel centre (region of lower concentration) comes into play and serves as a counteracting force. In normal elution mode, these two opposite forces balance each other, and a steady-state distribution of analyte is established with the largest particles at the accumulation wall and the shorter ones toward the centre of the channel. This is because as the size of the particles reduces, the diffusion coefficient increases, so that smaller particles diffuse to a greater extent into the high-speed zone compared to larger particles and are therefore eluted earlier [20–22].

The goal of this paper is to demonstrate that AF4 with UV–Vis and DLS detectors can provide a satisfactory assessment of the degree of polydispersity of the CB dispersions and their stability in a variety of culture cell media with or without amino acids. Real culture media used for human mesenchymal stem cell culture were also tested to confirm the dispersibility of CB-NPs. This accurate characterization, as mentioned above, is critical for extracting good results from in vitro toxicity assays in order to understand differences in the effective dose delivered to cells. Finally, the results demonstrate the application of this technique to in vitro toxicity tests for studying the viability of lung and liver cells after 48 h exposure to several concentrations of CB-NPs.

## Experimental

### Reagents and materials

CB powdered sample (grade N326) was obtained from Birla Carbon S.L. (Cantabria, Spain). Some CB properties provided by the manufacturer are detailed in Table S1 of the supporting information (SI). Tween 80 was supplied by Sigma-Aldrich (St. Louis, MO, USA), and Triton X-100 by Thermo Fischer Scientific (Waltham, MA, USA). All cell culture media used are listed in Tables S2 and S3 of the SI with suppliers and with their compositions, respectively. The cell culture media used for growing cells and in vitro toxicity assays are given in Tables S2 and S4 of the SI, respectively.

Also, L-glutamine BioUltra (≥ 99.5%) and sodium bicarbonate ACS reagent (≥ 99.7%) were purchased from Sigma-Aldrich (Table S5 shows the composition of stock solutions used for preparation of the amino acid-based dispersants).

The AF4-UV–Vis-DLS liquid carrier (0.1 μm filtered) was prepared with 0.02% NaN_3_ from Panreac (Barcelona, Spain). Water/methanol (VWR, Radnor, PA, USA) in an 80:20 ratio mixture was used for cleaning the AF4 system. Water for all the experiments was purified using a Sybron Barnstead NANOpure II system (ρ = 18.2 MΩ cm; total organic carbon < 2 parts per billion [ppb]).

### Instrumentation

The AF4 system used was an AF2000 MultiFlow FFF (Postnova Analytics GmbH, Landsberg am Lech, Germany) coupled online to an SPD-20AV UV–Vis detector (Shimadzu Corporation, Japan) and a Zetasizer Nano ZS DLS detector (Malvern, UK). Flows were provided by two separate PN1130 isocratic pumps (Postnova Analytics GmbH) equipped with a PN7520 degasser, and the cross-flow was obtained by a separate piston pump, which is constantly adjustable. The DLS detector can work in batch mode as well. Table S6 and S7 of the SI summarize the optimal conditions for batch DLS measurements and the optimal instrumental variables and conditions of the AF4 system for the study of CB-NP dispersions, respectively.

The morphology was studied by scanning electron microscopy (SEM) with a Hitachi S-4800 instrument at an accelerating voltage of 10.0 keV over metallized CB solid samples with a mixture of gold and palladium for 30 s. Transmission electron microscopy (TEM) samples were prepared by delivering 10 μL of the CB dispersion onto a carbon-coated copper grid (300 mesh) and were dried overnight at room temperature. These samples were analysed using a JEM-1010 (JEOL Ltd.) operated at 100 kV.

### Preparation of CB dispersions

Stock CB-NP dispersions were prepared by adding 2 mg of CB nanopowder sample (grade N326) to 10 mL of the corresponding dispersant (200 μg mL^−1^), followed by 2 h of ultrasonication in a water bath (see Fig. S1 of the SI for establishing this time), at room temperature in air atmosphere and stored at 2 °C for conservation. For the CB study on cell culture media-based dispersants, these were prepared by diluting 1× culture media (detailed in Tables S2 and S3) to 0.1× and adding a proper amount of Tween 80 to achieve a concentration of 2.0%.

For application to real samples, human mesenchymal stem cells were obtained from two biopsies of dental pulp from two volunteers and cultured in a 5% CO_2_ atmosphere at 37 °C. CB dispersions were prepared by adding 2 mg of CB nanopowder to 10 mL of the dispersant, followed by 2 h of ultrasonication. These culture media contained 10% fetal bovine serum (FBS, HyClone, USA) and 1% penicillin–streptomycin (Thermo Fisher, USA) necessary for cell culture.

Cel 6 cell culture media (summarized in Tables S2 and S4), which is similar to Cel 5 1×, was also supplemented with 10% FBS used in the presence of cells and prepared at 200 μg mL^−1^ of CB, and a proper amount of Tween 80 was added to achieve a 2.0% concentration.

For the CB study on amino acid-based dispersants, these were prepared by diluting the stock solutions detailed in Table S5 of the SI in a 1:10 ratio and adding a proper amount of Triton X-100 to achieve a concentration of 1.0%.

Linearity was evaluated from CB-NP dispersions in Cel 5 up to 200 μg mL^−1^, and the limit of detection (LOD) was calculated as the concentration of CB-NPs giving a signal-to-noise ratio (s/n) of 3 from fractograms obtained at 450 nm. Precision was evaluated by calculating the percentage relative standard deviation (RSD).

### In vitro toxicity assay

#### Cell culture and treatment

Human lung epithelial cell line (H1975) and liver cell line (HepG2) were obtained from the American Type Culture Collection (ATCC^®^); H1975 cells were cultured in Roswell Park Memorial Institute (RPMI) 1640 medium and HepG2 cells were cultured in high-glucose Dulbecco’s modified Eagle medium (DMEM) (see Tables S2 and S4 of the SI), both supplemented with 10% FBS and 1% penicillin–streptomycin in a 5% CO_2_ atmosphere at 37 °C. CB was heated at 200 °C for 120 min to sterilize, then resuspended by sonication in complete RPMI and DMEM medium with 2% Tween 80 at concentrations of 50, 100, and 200 μg mL^−1^. The working solutions were freshly diluted 40 times in complete RPMI and DMEM media to concentrations of 1.25, 2.5, and 5 μg mL^−1^. H1975 and HepG2 cells were treated with CB for 48 h. RPMI and DMEM medium with 0.05% Tween 80 was used as control.

#### Cell viability and LDH detection assay

Cell viability of lung and liver cells was studied using a CCK-8 (Cell Counting Kit-8) and MTT (3-(4,5-dimethylthiazol-2-yl)-2,5-diphenyltetrazolium bromide) assay. Both cell lines were seeded at 3 × 10^4^ cells/well in a 96-well plate. The next day, the cells were incubated with different concentrations of CB for 48 h. The CCK-8 assay was developed by adding 10 μL of the CCK-8 solution to each well. Cells were then incubated at 37 °C for 2 h, and the optical density of the cultures was measured at 450 nm. MTT reduction assay was developed by adding 0.5 mg mL^−1^ of MTT solution to each well for 2 h. Intracellular formazan was released with dimethyl sulfoxide (DMSO), and absorbance was measured at 550 nm. The results were expressed as a percentage of the control. After 48 h, the supernatant was collected and tested for lactate dehydrogenase (LDH) using the Cytotoxicity Detection Kit^PLUS^ (LDH) (Roche, Indianapolis, IN, USA). All absorbance measurements were performed using a Halo LED 96 spectrophotometer (Dynamica Scientific Ltd., Livingston, UK). All experiments were carried out in triplicate with at least three replicates.

## Results and discussion

### CB sample characterization

**Fig. 2:**
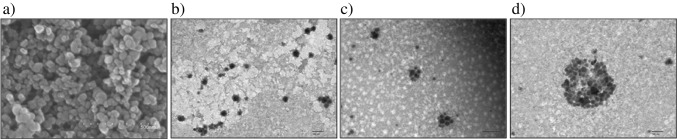
**a** Micrographs of grade N326 CB-NPs obtained for a solid bulk sample by means of SEM, and **b**–**d** for an aqueous dispersion in culture media Cel 1 by TEM. For further explanation, see text and supporting information

CB bulk sample (grade N326) and dispersed CB were measured by SEM and TEM, respectively. Electron microscopy results show a mean core size of 41 nm for the powder (see Fig. 2a), and as shown in Fig. 2b–c for aqueous dispersions, different spherical and ellipsoidal primary particles bind together to form aggregates with different linear, spherical, and elliptical structural forms. Meanwhile, in Fig. 2d, a large spherical agglomerate approximately 500 nm in size can be observed, which was formed by the reversible union of several aggregates to reduce surface hydrophobicity.

### Optimization of AF4 conditions for assessment of CB dispersions

In order to extract the desired information and to achieve good separation and characterization ability, proper selection of the working conditions is necessary. This is intended to avoid alterations in the separation and sample nature, including steric transition, aggregation, or other undesirable phenomena such as excessive particle–membrane interaction, as well as low retention that would call into question the representativeness of the results.

**Fig. 3:**
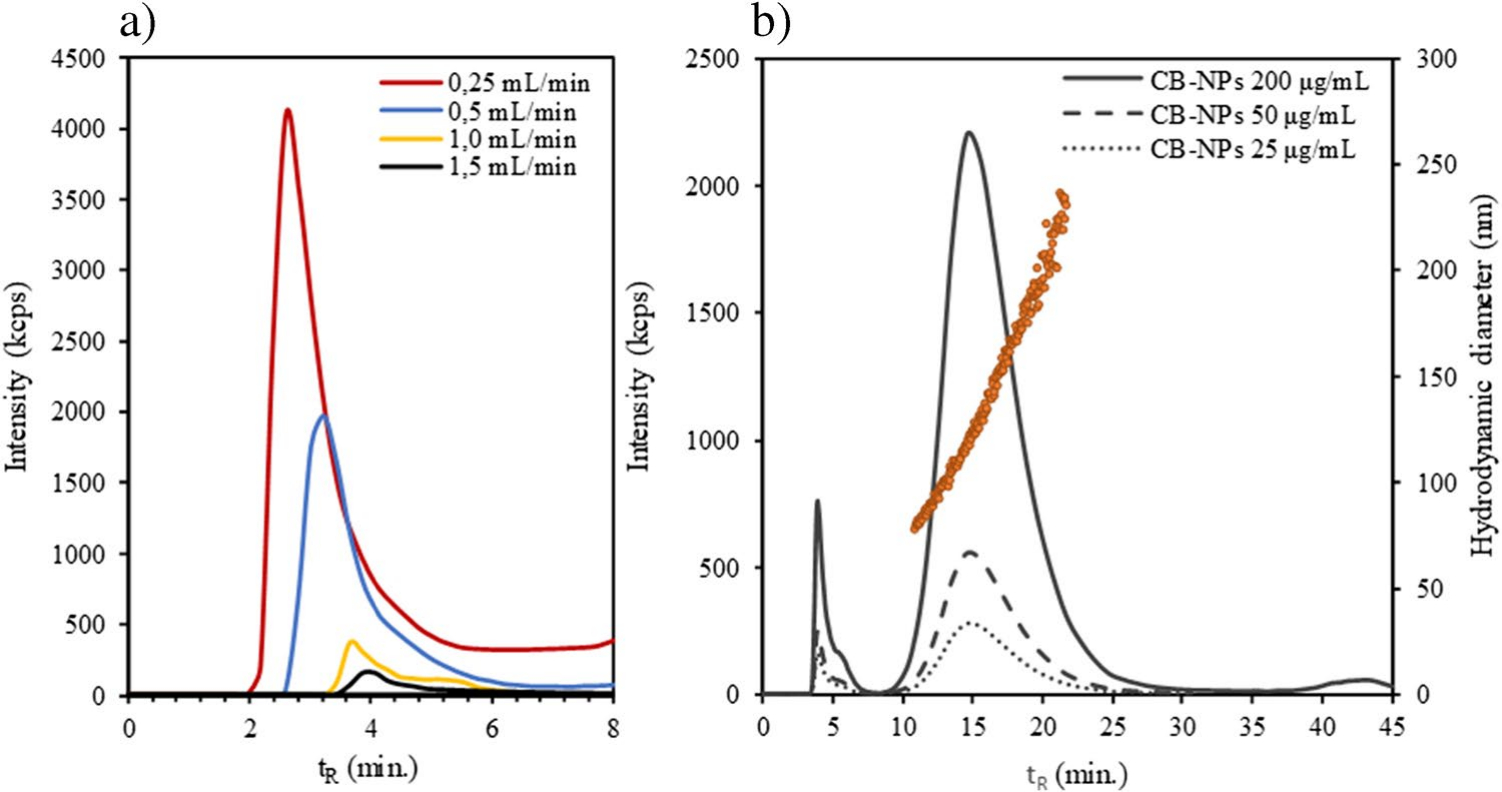
**a** Void peak corresponding to CB-NP N326 fractograms (200 μg mL^−1^) with DLS detector at different cross-flow rates and **b** CB-NP N326 DLS fractograms at optimized cross-flow gradient for dispersions in culture media Cel 5 with different CB concentrations. For further explanation, see text and supporting information

There are a wide variety of parameters that can influence the quality of the separation: carrier liquid, focusing time or membrane type, among others [2, 12]. However, although they are important parameters, they do not have a key influence on the elution profile. In AF4, it is the cross-flow (C_F_) that generates the force field that allows separation, and it is therefore the main factor for achieving adequate elution of the sample components. As can be seen in Fig. 3a, cross-flow has a huge impact on the void peak area, as a flow rate that is too low gives rise to a large part of CB-NPs eluting at void time. A high flow rate could solve this problem but at the expense of generating excessive retention and peak broadening, so a customized cross-flow gradient is required to optimize CB-NP elution. Figure 3b shows CB N326 fractograms obtained with optimized cross-flow gradient (detailed in Tables S6 and S7 of the SI). A proper separation from the void peak, which has a small area (< 5% relative to main peak), was achieved as well as a Gaussian profile for the main peak and a good correlation (R^2^ = 0.98) between the hydrodynamic diameter and retention time (t_R_) along the peak according to the normal elution mode (separation in growing size). The response to CB concentration was linear (see Fig. 3b and Table S8 of the SI), and the average size of the CB dispersion was 133 nm, which indicated that aggregates of the primary particles, mainly spherical as shown in Fig. 2, were dispersed.

It should be noted that a third low-intensity peak was observed when the cross-flow dropped to zero (*t*_R_ ≈ 43 min), which corresponded to a mixture of different particles that were adsorbed onto the membrane and left it with the mobile phase at this time, and/or to the small fraction of CB undispersed agglomerates with sizes ranging within 0.5–1 μm (see Fig. 2d), which were not able to elute in the presence of cross-flow.

More information about optimization of the AF4 parameters is given in the supporting information (see Table S7). The *I*_F_ is the flow rate at which the sample is transported into the separation channel during the sample injection and focusing stages. As can be seen in Fig. S2 of the SI, low *I*_F_ (below 0.15 mL min^−1^) leads to a marked increase in the peak area of the void peak, which causes a reduction in the signal observed at the peak of the CB-NPs. However, above 0.15 mL min^−1^, no significant variation is observed in the profile of the fractograms. In addition, it should be noted that adjusting the *I*_F_/C_F_ ratio to 0.1 implies establishing the focus point at 3.3 cm from the injection site, which corresponds to the point of maximum amplitude of the separation channel [19], as schematically described in Fig. S3 of the SI. Likewise, conditions with an *I*_F_/C_F_ ratio greater than 0.1 result in the focus point being shifted towards the channel exit, thus reducing the distance over which separation occurs, which can lead to a loss of resolution.

The *F*_T_ is the duration of the focusing stage, during which the sample components are focused at the same point before starting to separate. This parameter must be adjusted appropriately, since a very low F_T_ may imply an insufficient approach, leading to excessive elution of the CB-NPs in the void peak, although it provides lower *t*_R_. On the other hand, an *F*_T_ that is too large may imply an excessive residence of the sample components in the separation channel, which could cause interactions with the membrane or other NPs, as well as higher *t*_R_. As can be seen in Fig. S4 of the SI, as the *F*_T_ increases, the void peak decreases, especially between 0.3 and 1.5 min. In addition, a reduction in peak width is noted for the larger *F*_T_. However, no significant improvement is observed in these parameters for an *F*_T_ greater than 1.5 min., although this implies higher *t*_R_. Therefore, an *F*_T_ of 1.5 min was selected as optimal as a compromise between an adequate approach and a shorter analysis time.

### CB dispersibility and stability on culture media-based dispersants

**Fig. 4:**
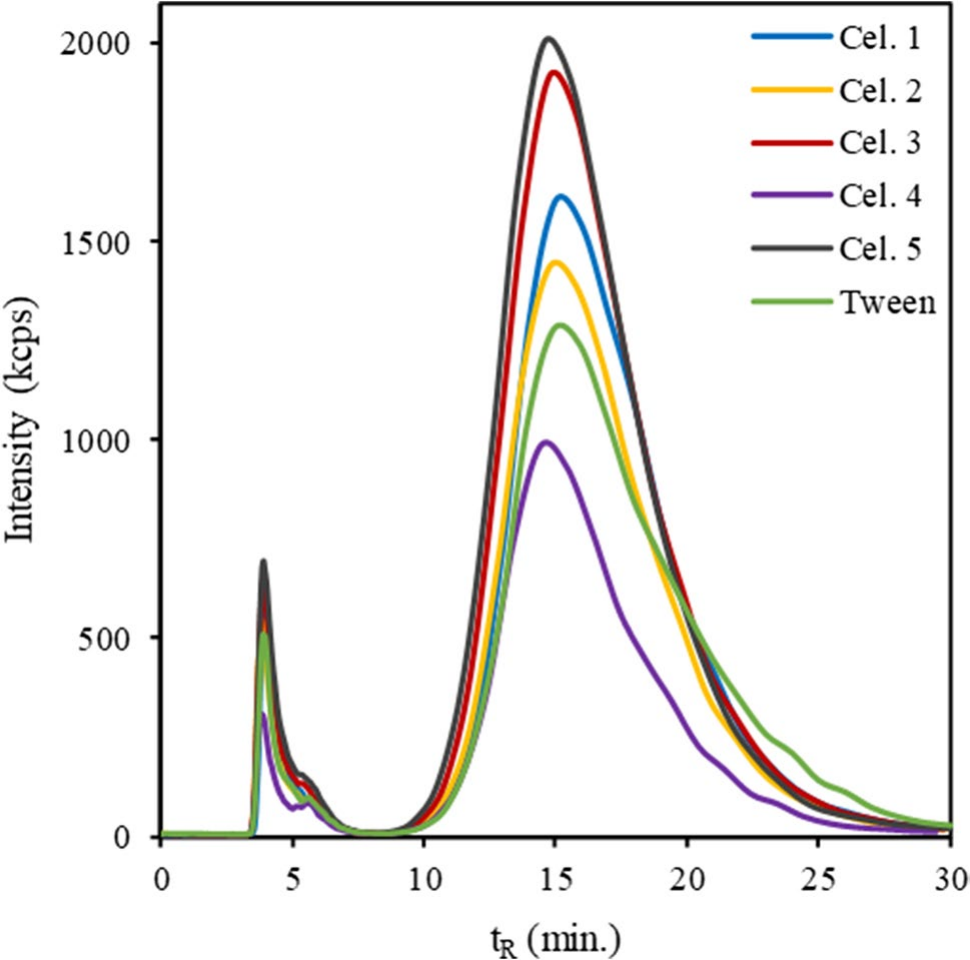
CB-NP N326 fractograms (200 μg mL^−1^) with DLS detector for the several types of cell culture media and Tween 80 as non-ionic surfactant. For further explanation, see text and supporting information

With the AF4-optimized conditions, CB-NP dispersibility was evaluated in different types of dispersants based on cell culture media as detailed in Tables S2 and S3 of the SI. Poor dispersibility and stability were obtained with culture media alone. Tween 80 (2.0%) has been used as non-ionic surfactant for all tested dispersants [12]. Surfactant alone provided poor stability as well. Fractograms of different CB-NP dispersions at the same concentration are shown in Fig. 4. As can be seen in this figure, the different culture media evaluated show significant differences. Except for the case of Cel 4 media, the use of cell culture media in combination with a non-ionic surfactant such as Tween 80 improved the dispersibility of CB-NPs, as can be deduced by the larger peak area observed in their fractograms. In terms of dispersibility, the best results were obtained for the Cel 5 medium, which in turn provided the smallest particle size, as shown in Table 1.

**Table 1:** Average CB-NP size estimated by both batch DLS and AF4-DLS, and zeta potential from batch DLS and size at the retention time of the AF4 peak apex for different cell culture media-based CB-NP dispersions

	AF4-DLS		Batch DLS	
Dispersant	Average size (nm)	Peak apex size^a^ (nm)	Average size^b^ (nm)	Zeta potential^c^ (mV)
Tween	159	133 ± 3	212 ± 12	−0.2 ± 0.2
Cel 1	139	120 ± 3	170 ± 6	−9.4 ± 0.7
Cel 2	145	125 ± 3	175 ± 9	−9.5 ± 0.7
Cel 3	145	126 ± 4	176 ± 10	−9.1 ± 0.3
Cel 4	157	135 ± 3	196 ± 11	−1.4 ± 0.1
Cel 5	133	117 ± 3	174 ± 5	−9.3 ± 0.6

^a^ Confidence interval (α = 0.01) (*n* = 10); ^b^ confidence interval (α = 0.01) (*n* = 3); ^c^ (*n* = 3)

Nevertheless, although dispersibility is important, the main problem that CB-NP dispersions have is their stability over time, since they generally have a tendency to rapid form large agglomerates that settle, which substantially reduces their applicability for in vitro assays and degrades their properties and characteristics. In this respect, the stability of the different cell culture media-based CB-NP dispersions was assessed. The sedimentation process was followed by the peak area loss over time observed in fractograms. Therefore, only the dispersed CB-NP fraction is considered, since non-dispersed CB is deposited, and those agglomerates that may remain are adequately separated in the fractogram due to their large size. Note that the sizes estimated by batch DLS (Table S6 of the SI) were larger than those provided by AF4 with DLS detection (see Table 1), which can indicate the level of polydispersity of the dispersions, as the batch DLS technique gives global information because it is not a separation technique as AF4 is.

**Fig. 5:**
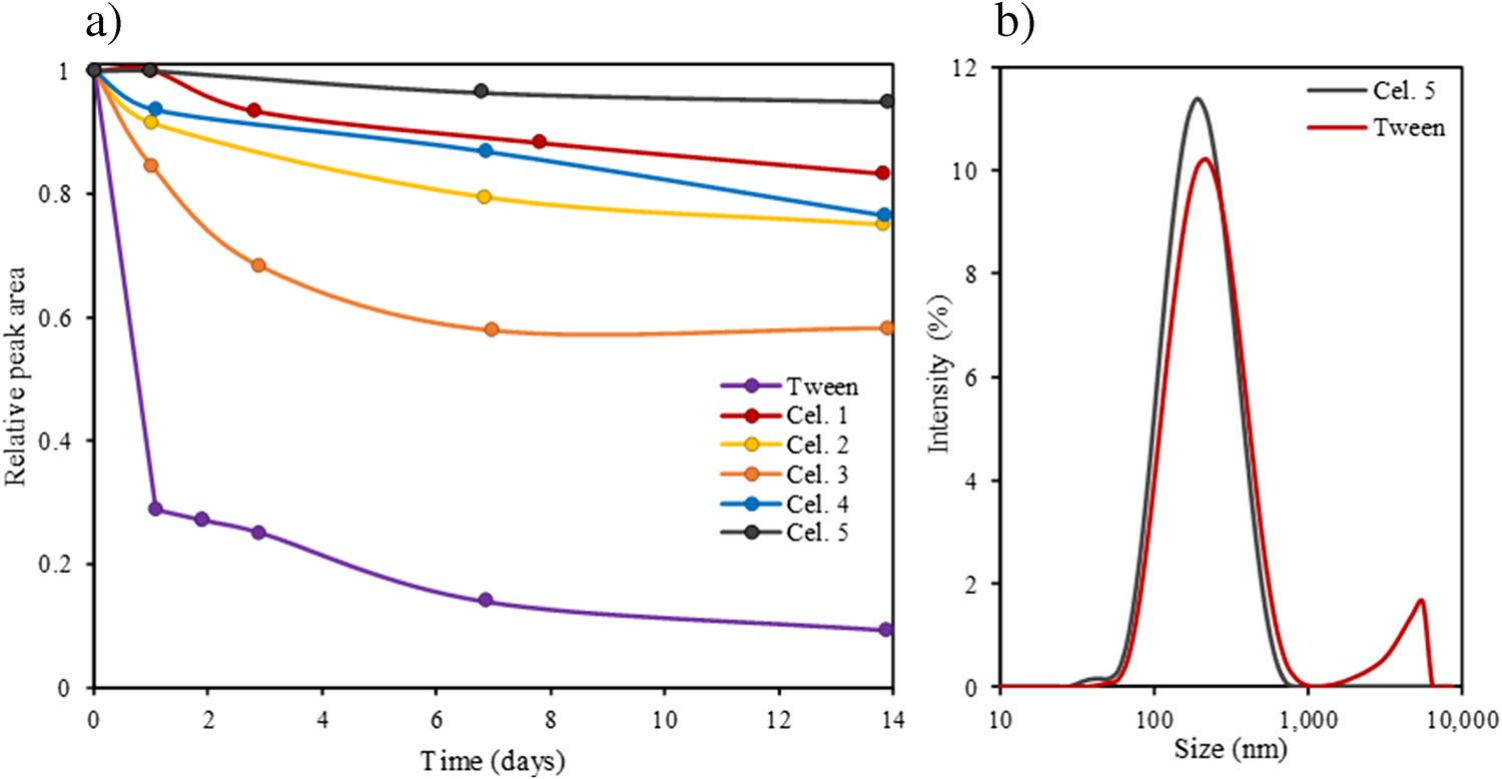
**a** Relative peak area evolution over time for the different cell culture media-based CB-NP dispersions (200 μg mL^−1^) and **b** size distribution of CB-NP dispersions in different types of dispersants obtained by batch DLS

Figure 5a shows the relative fractogram peak area evolution over time for each of the different dispersions. As can be seen, the dispersant without cell culture media offers the worst stability, reducing its peak area by more than 70% within just 1 day and more than 90% in 2 weeks. The use of non-ionic surfactants such as Tween 80 to stabilize CB-NP dispersions is based on the adsorption onto the surface of CB aggregates through their hydrophobic part and the presence of polar ethylene oxide polymers that provide a steric stabilization effect.

Hence, to prevent CB particle agglomeration it is necessary to incorporate some additional energy barrier besides the steric stabilization layer provided by the non-ionic surfactant. In this context, it would be suitable to add an electrostatic potential barrier to CB-NPs in order to prevent particles from approaching and adhering to one another. The size of this potential barrier can be estimated by the zeta potential magnitude, which is the potential at the slipping plane between the electrical double layer with the surrounding solvent [23]. In this respect, it was observed that the adsorption of this kind of surfactant to CB causes a zeta potential very close to zero and near that provided by Cel 4, as shown in Table 1. These data can explain its lower dispersibility than that provided by the other dispersants. As can be seen in Table 1, the zeta potential values for the other dispersants are similar and are more negative; however, different levels of dispersibility were achieved in the order Cel 5 > Cel 3 > Cel 1 > Cel 2, which can be explained by the different CB corona composition.

Nevertheless, the use of cell culture media shows a significant improvement in stability, preserving more than 90% of the peak area within 1 day and around 80% in 2 weeks, except for the case of Cel 3, which presented values of 85% and 60%, respectively. This stability enhancement can be understood as the use of cell culture media leading to more negative zeta potentials, which generates a greater electrostatic repulsion that prevents CB from forming agglomerates and results in greater stability of CB-NP dispersions.

This is clearly observed in Fig. 5b obtained from batch DLS, where two populations of CB-NPs can be distinguished for the dispersant without cell culture media: one centred around 100–200 nm, corresponding to the main dispersed fraction in the form of aggregates, and another of larger size centred between 4 and 5 μm that corresponds to CB agglomerates, which cannot be prevented from settling by the dispersant. On the contrary, for dispersant Cel 5, which provided the best results, a single population of dispersed CB-NPs with a smaller average size can be observed.

Regarding cell culture media composition, there are some similarities between them, such as basic pH, a high-ionic-strength environment, or the presence of certain biomolecules like vitamins or proteins. Nevertheless, there are also differences including the presence or absence of substances such as glucose, HEPES buffering, amino acid content, and the amount and variety of inorganic salts.

The total amount of amino acids can be an important parameter in cell culture media for explaining the achieved results, since it seems that dispersants with a low amino acid content, such as Cel 4, had worse CB-NP dispersal, giving rise to a smaller peak area and lower zeta potential magnitude, as observed in Fig. 4 and Table 1, respectively. This is different from the trend observed for other cell culture media, such as Cel 5, which provided the best results and had the highest total amino acid content.

Likewise, it should be noted that biomolecules such as amino acids are zwitterionic species that have easily ionizable functional groups that can modify the electrostatic properties of the NP surface. Furthermore, CB has particularly good biocompatibility in different biological media [10], and other studies have shown that the presence of biomolecules such as proteins can improve the stability of CB-NP dispersions [24]. To confirm this hypothesis, principal component analysis (PCA) was applied considering the amounts of selected compounds in the several culture media assayed (see Tables S2 and S3 of the SI) and the size of the CB in the obtained dispersions.

**Fig. 6:**
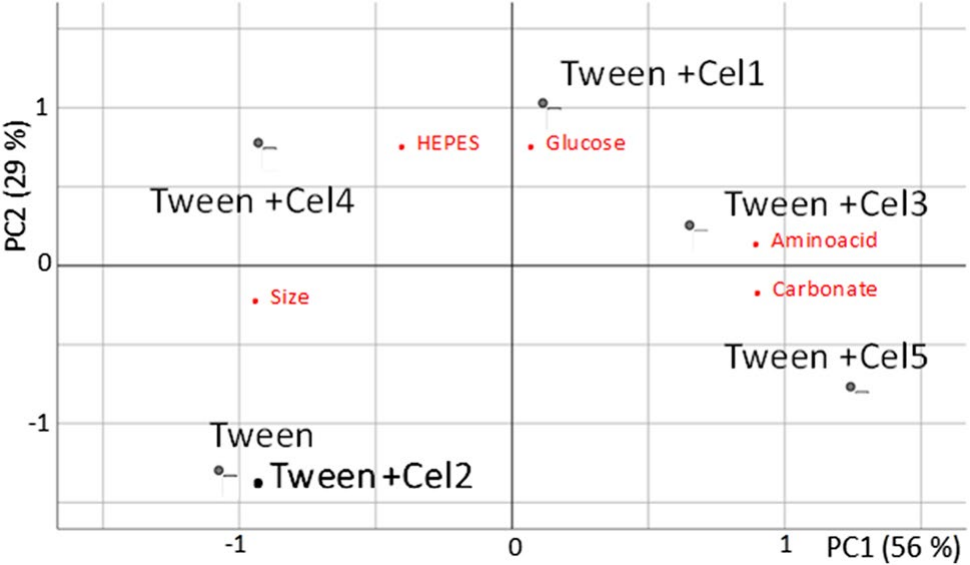
Principal component analysis: biplot showing the loadings of variables in principal components and scores of the studied dispersants. For further explanation, see text and supporting information

Figure 6 shows a biplot of PCA, and the explained variance with two PCs was good, at 85%. Two groups can be observed with respect to the first PC. Note that carbonate and amino acids are inversely related in size, bearing in mind that their loadings and the three variables are the most important for the first PC. The results achieved are in accord with previous statements (see Fig. 5). As Cel 2 was not contained or its content was lower than that corresponding to the other cell culture for the selected compounds, the score obtained for it was similar to that corresponding to Tween alone, and the size of the CB was the most important variable that explained both scores. For Cel 4 and Cel 1, the scores were more determined by HEPES content and glucose, respectively. Cel 3 and Cel 5 scores were related to carbonate and amino acid content. However, Cel 3 provided good dispersibility, but stability was worse than that achieved by Cel 5. Cel 3 composition differed with respect to Cel 5 in the amount of amino acids, which was lower, and the presence of glucose.

### Study of amino acid influence on CB dispersion

In view of the results obtained with cell culture media-based dispersants, several aqueous media with different amino acid content were prepared. The media composition is detailed in Table S5 of the SI. A variable concentration of amino acids was studied, with L-glutamine being selected as the model amino acid as it is the most abundant in different culture media. A constant amount of sodium bicarbonate was added in order to match the cell culture media pH range. No other inorganic salt was considered, since excessively high ionic strength could screen surface charge, giving rise to a lower zeta potential magnitude than expected and masking the electrostatic stabilization previously observed. Triton X-100 was studied as the surfactant instead of Tween 80 in order to assay the influence of other non-ionic surfactants.

**Fig. 7:**
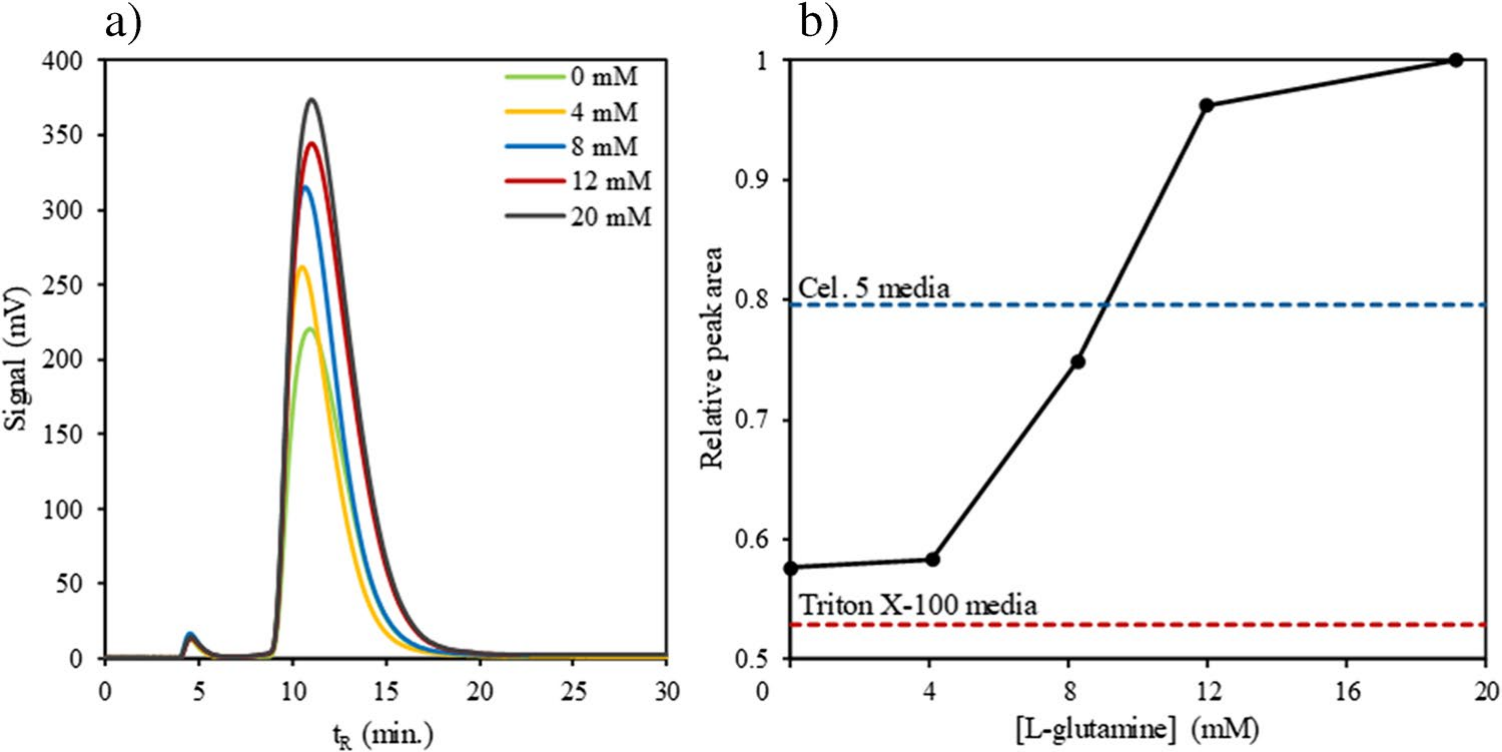
**a** Fractograms of CB-NP N326 (200 μg mL^−1^) dispersions with UV–Vis detector at 450 nm in several media with different L-glutamine concentrations and constant amounts of Triton X-100 as non-ionic surfactant and bicarbonate; **b** relative peak area evolution with L-glutamine concentration for the different amino acid-based CB-NP dispersions including the results obtained for Triton X-100 alone and the results for dispersions containing Tween and Cel 5 studied in the previous section

**Fig. 8:**
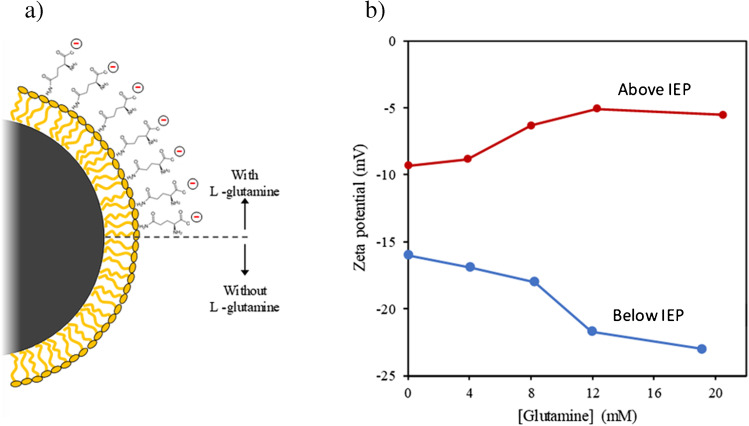
**a** Scheme of the CB corona with surfactant alone and in the presence of L-glutamine and **b** zeta potential tendency of amino acid-based CB-NP dispersions with L-glutamine concentration above and below its isoelectric point (IEP)

As shown in Fig. 7a, b, there is a clear relation between the CB-NP peak area and amino acid content of the dispersing media. Thus, under assayed conditions, it seems that amino acids tend to adsorb onto the CB surface, and hence, in addition to the surfactant, they become part of the particle corona as Fig. 8a illustrates. Other works have already shown the ease with which carboxyl and amino compounds attach to the NP surface, since primary amino/carboxyl groups are major determinants in the binding of organic compounds to the particles [14]. The behaviour of the Triton X-100 was similar to that of Tween 80.

Taking this into account, since amino acids are zwitterionic species with at least two easily ionizable protic groups, it is possible to alter the surface electrostatic properties of CB-NPs by changing the media pH above or below the IEP. Thus, new amino acid-based CB-NP dispersions were prepared by adjusting dispersant pH above (pH 8.8) and below (pH 3.7) L-glutamine IEP (pH 5.56), and the zeta potential was then measured, with results shown in Fig. 8b.

As can be seen in Fig. 8b, the trend of CB-NP zeta potential with L-glutamine concentration is the opposite under acidic and basic conditions. In acidic media, the pH is below the IEP; hence, the L-glutamine positive ions are the predominant form, so the average net charge is positive. Thus, as L-glutamine adsorbs to the CB surface, the zeta potential becomes less negative, which decreases the electric potential barrier, and therefore reduces CB-NP stability. Conversely, in basic media, the pH is above the IEP; hence, the L-glutamine negative ions are the predominant form, so that in this case, the average net charge is negative. Thus, L-glutamine adsorbed to the CB surface turns the zeta potential more negative, which increases the electric potential barrier, and consequently enhances CB-NP stability in aqueous media.

Table 2 gives general values with respect to colloid stability as a function of zeta potential in absolute values. CB-NP dispersions based exclusively on non-ionic surfactants are in the range of strong agglomeration and precipitation (0 to −5 mV), whereas amino acid-based CB-NP dispersions with optimized composition allow this magnitude to increase to close to the moderate stability range (< −30 mV) [23].

**Table 2:** Colloid stability behaviour dependence on zeta potential for electro-stabilized dispersions [23]

Zeta potential (mV)	Colloid behaviour
0–5	Rapid coagulation or flocculation
10–30	Incipient instability
30–40	Moderate instability
40–60	Good stability
> 61	Excellent stability

All in all, this study demonstrates the importance of NP corona composition on the dispersibility and stability of hydrophobic particles such as CB in aqueous media. Both steric and electrostatic stabilization are required to obtain high-quality aqueous dispersions for highly unstable hydrophobic colloids. This implies that the kind of culture media selected will influence the in vitro toxicity assays for CB assessment. The pH of the assay is another important factor, and the physiological pH will be favourable considering the isoelectric point of glutamine, as the most abundant amino acid in these culture media.

**Fig. 9:**
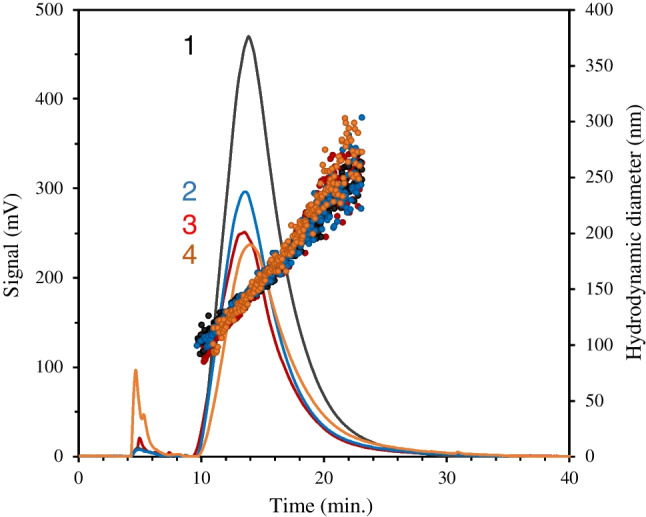
CB-NP N326 fractograms (200 μg mL^−1^, UV–Vis detector at 450 nm) for Cel 6 media with (trace 1) and without 10% FBS (trace 3) and two real culture media Cel 6 used for cell culture of two biopsies of two volunteers (traces 2 and 4) after 48 h in a 5% CO_2_ atmosphere at 37 °C, which contained 10% FBS and 1% penicillin–streptomycin

The dispersion of CB-NPs in culture media used for cell culture was tested, and the results are shown in Fig. 9. All peaks presented an average size of 133 nm for the different CB-NP dispersions tested, the same as that obtained with Cel 5 (see Table 1). The Cel 6 culture media used is similar in composition to Cel 5, and the amount of amino acids is the same, with the same concentration of L-glutamine (see Tables S3 and S4 of the SI). The CB-NP dispersion was prepared in Cel 6 as in optimized conditions for Cel 5. Other dispersion supplemented with 10% FBS, which was used in cell culture, was measured and also the CB-NP dispersion from two culture media that were in contact with human mesenchymal stem cells for 48 h in a 5% CO_2_ atmosphere at 37 °C obtained from two biopsies of two volunteers.

As we can seen in Fig. 9, a signal enhancement was obtained by adding FBS to the culture media (black line) with respect to the CB-NP dispersion obtained from Cel 6. FBS is a complex mixture of biomolecules, important for the growth and maintenance of cells in vivo and in culture, which includes growth factors: proteins, trace elements, vitamins, and hormones. The presence of the supplemented proteins from FBS can explain the higher dispersibility of the CB-NPs relative to that obtained with Cel 6 not supplemented.

On the other hand, the fractograms showed slightly higher areas for the two real culture media Cel 6 used for the cell culture of two biopsies of two volunteers (traces 2 and 4 of Fig. 9) than those obtained from the dispersion in Cel 6 (trace 3 of Fig. 9), which indicated good dispersibility in real situations. However, these areas were smaller for the culture media in contact with cells in a 5% CO_2_ atmosphere at 37 °C in comparison with that achieved by Cel 6 supplemented with 10% FBS at room temperature and in air atmosphere. The real media also contained 10% FBS and 1% penicillin–streptomycin. This phenomenon is most likely due to the consumption of growth factors by the cells or/and cell culture conditions. In any case, this fact suggests that the in vitro toxicity tests should consider a blank assay without cells.

### In vitro toxicity tests of CB-NPs

**Fig. 10:**
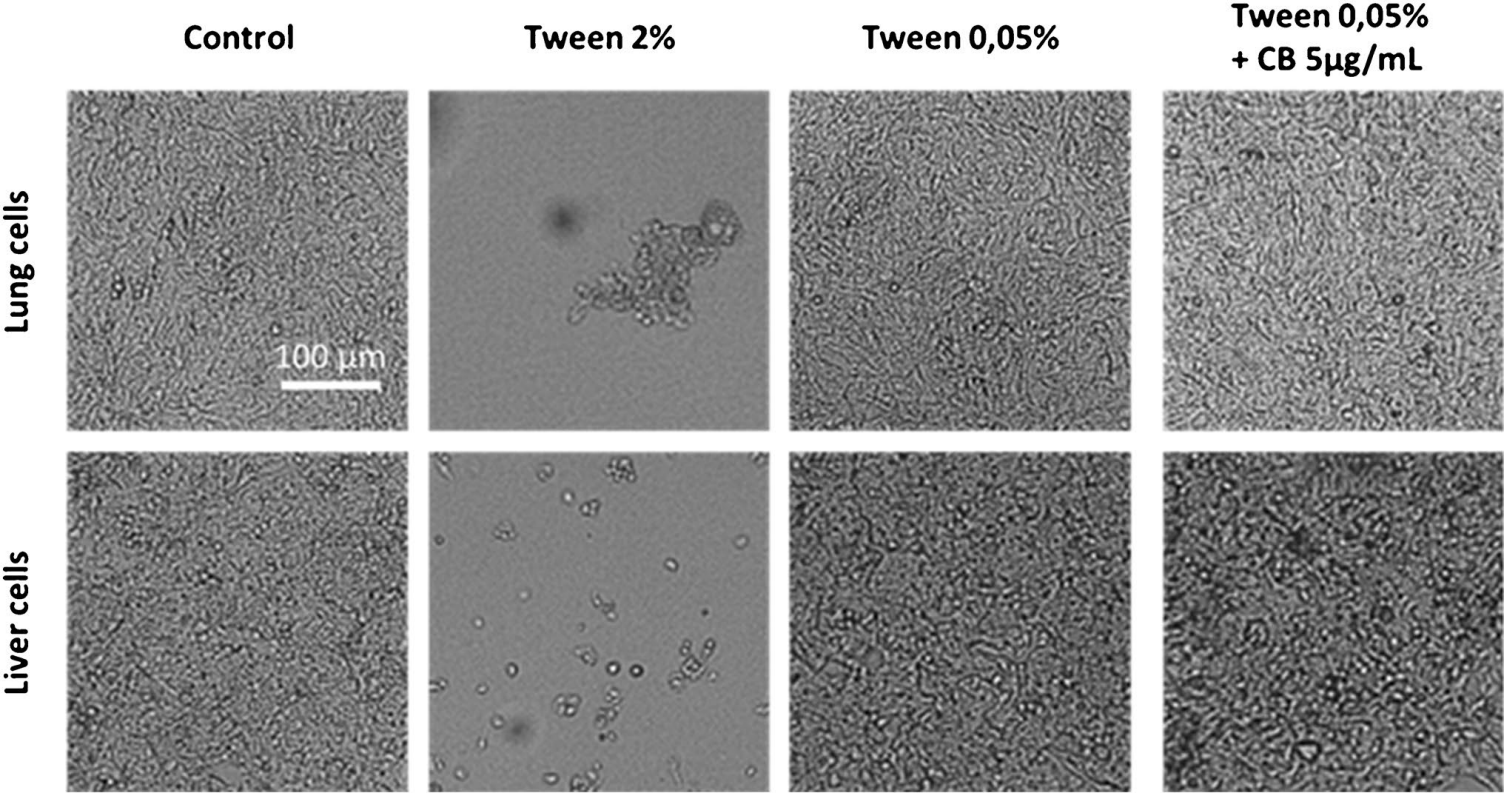
Effect of Tween 80 in culture cells. Representative bright-field microscopy images of lung and liver cells after 48 h of culture. Cells were cultured on completed medium (control), with completed medium supplemented with Tween 80 (2% or 0.05%) and treated with 5 μg mL^−1^ of CB diluted in completed medium with 0.05% of Tween 80. Scale bar: 100 μm

CB was resuspended in the culture medium with 2% Tween 80, as indicated in the experimental section. However, Tween 80 is a detergent that can be toxic to cells at some concentrations because it is able to degrade the cell membrane in a short time, as we show in Fig. 10. In order to use CB dispersions for cell culture, a 40-fold dilution was established to reduce the amount of Tween 80 to cell-viable concentrations (0.05%). We observed that the appearance of the cells in this culture is the same with Tween-free medium as with medium at low Tween 80 concentrations.

**Fig. 11:**
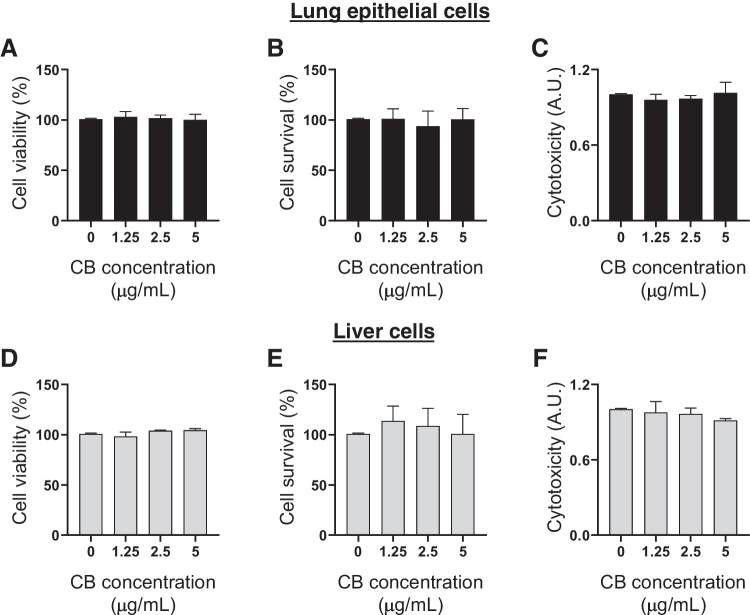
Effects of CB on cell viability and cytotoxicity. The viability of lung and liver cells after 48 h exposure to low concentrations of CB. Cell viability was measured by CCK-8 (**A** and **D**) and MTT (**B** and **E**) assay and expressed as percentages of the control. (**C** and **F**) Relative quantification of lactate dehydrogenase (LDH) activity measured by the absorbance in lung and liver cells after 48 h exposure to low concentrations of CB. The bars represent the mean and standard deviation (SD) of three independent experiments. Cells were exposed to 0, 1.25, 2.5, and 5 μg mL^−1^ of CB

Lung epithelial and liver cells were treated with different concentrations (1.25, 2.5, or 5 μg mL^−1^) of CB for 48 h. First, we observed that there were no apparent significant differences between the control (Tween 80 0.05%) and CB-treated cells, even at the highest concentration (5 μg mL^−1^). Then, CCK-8 and MTT assays were employed to evaluate the viability of cells. The results of CCK-8 assay (Fig. 11a and d) demonstrated that the cell viability of lung and liver cells was not affected in response to exposure to CB. The results of MTT assay (Fig. 11b and e) also confirmed that cell survival remained the same as the control in the CB exposure conditions. The effect of CB on cytotoxicity in lung and liver cells was evaluated by measuring the presence of lactate dehydrogenase (LDH) after exposure to CB (1.25, 2.5 or 5 μg mL^−1^) for 48 h. The level of LDH production also showed that the CB exposure concentration had no effect on cytotoxicity (Fig. 11c and f).

## Conclusions

In this work, a method was developed for the study, characterization, and quantification of CB-NPs dispersed in culture media by means of the AF4 technique, optimizing parameters such as cross-flow gradient or focusing step, where cross-flow proved to be the key parameter to ensure adequate elution.

A study was carried out to evaluate the dispersibility and stability of CB-NPs in cell culture media-based dispersants and identify compositional variables that favour CB dispersal. The results obtained show that culture media with a higher amino acid content have better dispersive ability and provide improved stability. These media generated smaller particles, as they are able to counteract CB-NP surface hydrophobicity and prevent their agglomeration. Zeta potential measurements show an increase in zeta potential magnitude with amino acid concentration, so that a new colloidal stabilization mechanism besides steric effect comes into play.

The influence of total amino acid content on CB-NP aqueous dispersion quality was studied. The results show a clear improvement in media dispersibility with amino acid concentration, so it seems that amino acids tend to adsorb onto the CB surface and, in addition to the surfactant present in the dispersion needed also for achieving the mentioned stability, they become part of the particle corona. Since they are attached to CB, it is possible to alter the surface charge by modifying dispersant pH above and below the IEP, giving rise to more negative zeta potential values.

The results achieved were corroborated in real situations, with culture media used for human mesenchymal stem cells cultured from dental pulp from two biopsies of two volunteers. This accurate characterization, as mentioned before, is critical for extracting good results from in vitro toxicity assays for understanding differences in the effective dose delivered to cells. Finally, the method was applied to in vitro toxicity tests for studying the viability of lung and liver cells after 48 h exposure to several concentrations of CB-NPs (1.25, 2.5 or 5 μg mL^−1^). The cell viability of lung and liver cells was not affected in response to exposure to CB. Cell survival remained the same as the control in the CB exposure conditions, and these CB concentrations had no effect on cytotoxicity.

## Supplementary Information

Below is the link to the electronic supplementary material.

Supplementary file1 (DOCX 245 kb)
