# Supplementary material for: Reliable assessment of carbon black nanomaterial of a variety of cell culture media for in vitro toxicity assays by asymmetrical flow field-flow fractionation
Source: Anal Bioanal Chem. 2023 Feb 25;415(11):2121–32. doi: 10.1007/s00216-023-04597-8 (PMC10079754; doi:10.1007/s00216-023-04597-8)
Supplement: Supplementary file 1 — Supplementary file1 (DOCX 245 kb) [file 216_2023_4597_MOESM1_ESM.docx]

**Reliable assessment of Carbon Black nanomaterial on a variety of cell culture media for in-vitro toxicity assays by asymmetrical flow field-flow fractionation**

A. Boughbina-Portolés^1^, L. Sanjuan-Navarro^1^, L. Hakobyan^2^, M. Gómez-Ferrer^2^, Y. Moliner-Martínez^1^, P. Sepúlveda^2^, P. Campíns-Falcó^1.^*

^1^MINTOTA research group. Departament de Química Analítica. Facultat de Química. Universitat de València. Dr. Moliner 50. 46100-Burjassot. Valencia. Spain.

^2^*Regenerative Medicine and Heart Transplantation Unit. Instituto de Investigación Sanitaria la Fe. Avda. Fernando Abril Martorell 106. 46026 Valencia. Spain*

E-mail corresponding author: [pilar.campins@uv.es](mailto:pilar.campins@uv.es)

**Supporting Information**

**Table S1.** Primary particle size (PPS), surface area (NSA), oil absorption number (OAN) and density for CB grade N326 sample.

| Parameters | PPS (nm) | NSA (m^2^/g) | OAN (mL/100 g) | Density (kg/m^3^) |
| --- | --- | --- | --- | --- |
| CB N326 | 41 ± 2 | 78 | 72 | 455 |

**Table S2.** Supplier, reference and main description of the different cell culture media used in the work.

| Number | Supplier | Name | Description | Reference |
| --- | --- | --- | --- | --- |
| Cel 1 | Sigma-Aldrich | StableCell™ DMEM/F12 | With stable glutamine, 15mM HEPES and sodium bicarbonate, liquid sterile-filtered, suitable for cell culture. | D0697 |
| Cel 2 | Merck KGaA | DMEM/F-12 PLUS Basal Medium | The media contains high-glucose and does not contain L-glutamine or penicillin-streptomycin. | SCM162 |
| Cel 3 | Thermo Fischer Scientific | Advanced DMEM (1x) | The media contains high-glucose, non-essential amino acids, sodium pyruvate and phenol red. Does not contain L-glutamine. | 12491-015 |
| Cel 4 | Sigma-Aldrich | DMEM/Nutrient Mixture F-12 Ham | With 15 mM HEPES, without L-glutamine, L-leucine, L-lysine, L-methionine.,CaCl2. MgCl2, MgSO4, sodium bicarbonate. and phenol red. Powder. suitable for cell culture | D9785 |
| Cel 5 | Thermo Fischer Scientific | DMEM (1x) | The media contains L-glutamine and phenol red. Does not contain glucose, HEPES or sodium pyruvate. | 11966-025 |
| Cel 6 | Thermo Fischer Scientific | DMEM. low glucose. pyruvate | The media contains L-glutamine, phenol red, low-glucose and sodium pyruvate. Does not contain HEPES. | 31885-023 |
| Cel 7 | Thermo Fischer Scientific | DMEM. high glucose. pyruvate | The media contains L-glutamine, phenol red, high-glucose and sodium pyruvate. Does not contain HEPES. | 41966-029 |
| Cel 8 | Thermo Fischer Scientific | RPMI 1640 Medium | The media contains L-glutamine. phenol red and glucose. Does not contain HEPES or sodium pyruvate. | 21875-034 |

**Table S3.** Cell culture media composition provided by manufacturer.

|  | Cel 1 |  | Cel 3 | Cel 4 | Cel 5 |
| --- | --- | --- | --- | --- | --- |
| Inorganic salts (mM) | 140.8874431 |  | 162.3134926 | 124.8451492 | 162.4379788 |
| CaCl_2_ | 1.0509160 |  | 1.80206156 | — | 1.802061558 |
| CuSO_4_ • 5 H_2_O | 0.0000052 |  | — | 0.0000052 | — |
| Fe(NO_3_)_3_ • 9 H_2_O | 0.0001238 |  | 0.00024753 | 0.00012376 | 0.000247526 |
| FeSO_4_ • 7 H_2_O | 0.0014999 |  | — | 0.00149992 | — |
| MgCl_2_ • 6 H_2_O | 0.3010290 |  | — | — | — |
| MgSO_4_ | 0.4057570 |  | 0.81143098 | — | 0.811430983 |
| KCl | 4.1823550 |  | 5.36543293 | 4.18235497 | 5.36543293 |
| NaHCO_3_ | 14.2845919 |  | 44.0441584 | — | 44.04415844 |
| NaCl | 119.7067902 |  | 109.508785 | 119.70679 | 109.5087847 |
| Na_2_HPO_4_ | 0.5002860 |  | 0.78137649 | 0.500286 | — |
| NaH_2_PO_4_ | 0.4525867 |  | — | 0.45258675 | 0.905862744 |
| ZnSO_4_ • 7 H_2_O | 0.0015023 |  | — | 0.00150235 | — |
| Amino acids (mM) | 7.250562986 |  | 7.37622343 | 3.721421508 | 10.67304614 |
| L-Alanine | 0.050510719 |  | 0.09989898 | 0.049949489 | — |
| L-Alanyl-L-Glutamine | 2.497606114 |  | — | — | — |
| L-Arginine • HCl | 0.700180385 |  | 0.3987468 | 0.700180385 | 0.398746796 |
| L-Asparagine | 0.049956704 |  | 0.09990917 | 0.049956704 | 0 |
| L-Aspartic Acid | 0.049958681 |  | 0.09991736 | 0.049958681 | 0 |
| L-Cystine • 2 HCl | 0.099897835 |  | 0.20113658 | 0.056062831 | 0.201136581 |
| L-Cysteine • HCl • H_2_O | 0.099977226 |  | — | 0.178148486 | — |
| L-Glutamic Acid | 0.049955821 |  | 0.09991164 | 0.049955821 | — |
| L-Glutamine | — |  | — | — | 3.996168058 |
| Glycine | 0.249766884 |  | 0.49953377 | 0.249766884 | 0.399627015 |
| L-Histidine • HCl • H_2_O | 0.150169346 |  | 0.200353 | 0.150169346 | 0.200353003 |
| L-Isoleucine | 0.415262636 |  | 0.80048792 | 0.415262636 | 0.800487916 |
| L-Leucine | 0.450179157 |  | 0.80048792 | — | 0.800487916 |
| L-Lysine • HCl | 0.499589379 |  | 0.79934301 | — | 0.799343006 |
| L-Methionine | 0.115541854 |  | 0.20105891 | — | 0.20105891 |
| L-Phenylalanine | 0.214782977 |  | 0.39953992 | 0.214782977 | 0.399539924 |
| L-Proline | 0.149830626 |  | 0.09988708 | 0.149830626 | — |
| L-Serine | 0.249785898 |  | 0.4995718 | 0.249785898 | 0.399657436 |
| L-Threonine | 0.4487102 |  | 0.79752047 | 0.4487102 | 0.797520467 |
| L-Tryptophan | 0.044165891 |  | 0.07834304 | 0.044165891 | 0.078343045 |
| L-Tyrosine • 2 Na • 2 H_2_O | 0.213607474 |  | 0.39819282 | 0.213607474 | 0.398192817 |
| L-Valine | 0.451127178 |  | 0.80238325 | 0.451127178 | 0.802383249 |
| Vitamines (mM) | 0.183631604 |  | 0.169345669 | 0.183728087 | 0.159583857 |
| D-Biotin | 0.000014326 |  | — | 0.00001433 | 0 |
| Choline Chloride | 0.064317433 |  | 0.02864919 | 0.064317433 | 0.028649191 |
| Folic Acid | 0.006026280 |  | 0.00906208 | 0.00602628 | 0.009062075 |
| myo-Inositol | 0.069937833 |  | 0.03996448 | 0.069937833 | 0.039964476 |
| Niacinamide | 0.016541107 |  | 0.03275467 | 0.016541107 | 0.032754668 |
| D-Pantothenic Acid • ½Ca | 0.009401100 |  | 0.00838574 | 0.0094011 | 0.008385744 |
| Pyridoxal • HCl | — |  | — | 0.009822218 | 0 |
| Pyridoxine • HCl | 0.009876483 |  | 0.01945147 | 0.000150749 | 0.019451469 |
| Riboflavin | 0.000581890 |  | 0.00106281 | 0.00058189 | 0.001062812 |
| Thiamine • HCl | 0.006433442 |  | 0.01185888 | 0.006433442 | 0.011858879 |
| Vitamin B12 | 0.000501710 |  | — | 0.00050171 | 0 |
| Ascorbic Acid phosphate | — |  | 0.00976181 | — | 0 |
| D-Calcium pantothenate | — |  | 0.008394544 | — | 0.008394544 |
| Proteines (mg·L^-1^) | — |  | — | — | — |
| AlbuMAX® II | — |  | 400 | — | — |
| Human Transferrin (Holo) | — |  | 7.5 | — | — |
| Insulin Recombinant Chain | — |  | 10 | — | — |
| Other components (mg·L^-1^) | — |  | — | — | — |
| D-Glucose | 3150 |  | 4500 | 3150 | — |
| HEPES | 3574.5 |  | — | 3574.5 | — |
| Hypoxanthine | 2.44 |  | — | 2.1 | — |
| Linoleic acid | 0.042 |  | — | 0.042 | — |
| Phenol Red • Na | 8.63 |  | 15 | — | 15 |
| Putrescine • 2 HCl | 0.081 |  | — | 0.081 | — |
| Pyruvic acid • Na | 55 |  | 110 | 55 | — |
| DL-Thioctic Acid | 0.105 |  | — | 0.105 | — |
| Thymidine | 0.365 |  | — | 0.365 | — |
| Glutathione | — |  | 1 | — | — |
| Ethanolamine | — |  | 1.9 | — | — |

**Table S4.** Composition of cell culture media used for samples which contained cells and in-vitro assay.

|  | Cel 6 DMEM-low glucosa | Cel 7  DMEM-high glucose | Cel 8 (RMPI) |
| --- | --- | --- | --- |
| Inorganic salts (mM) | 163.2475425 | 163.2475424 | 139.0556603 |
| CaCl_2_ | 1.8018018 | 1.8018018 | — |
| Ca(NO_3_)_2_ • 4 H_2_O | — | — | 0.42372882 |
| Fe(NO_3_)_3_ • 9 H_2_O | 0.000247525 | 0.000247525 | — |
| MgSO_4_ | 0.8139166 | 0.8139166 | 0.407 |
| KCl | 5.3333335 | 5.3333335 | 5.3333335 |
| NaHCO_3_ | 44.04762 | 44.04762 | 23.809525 |
| NaCl | 110.344826 | 110.344826 | 103.44827 |
| NaH_2_PO_4_ • H_2_O | 0.9057971 | 0.9057971 | — |
| Na_2_HPO_4_ | — | — | 5.633803 |
| Amino acids (mM) | 10.67304614 | 10.67304614 | 6.561665191 |
| Glycine | 0.399627015 | 0.399627015 | 0.133209005 |
| L-Arginine • HCl | 0.398746796 | 0.398746796 | — |
| L-Arginine | — | — | 1.148105626 |
| L-Asparagine | — | — | 0.378443839 |
| L-Aspartic acid | — | — | 0.150251672 |
| L-Cystine • 2 HCl | 0.201136581 | 0.201136581 | 0.20752187 |
| L-Glutamic Acid | — | — | 0.135934208 |
| L-Glutamine | 3.996168058 | 3.996168058 | 2.052826057 |
| L-Histidine • HCl • H_2_O | 0.200353003 | 0.200353003 | — |
| L-Histidine | — | — | 0.096677765 |
| L-Hydroxyproline | — | — | 0.15267175 |
| L-Isoleucine | 0.800487916 | 0.800487916 | 0.381184722 |
| L-Leucine | 0.800487916 | 0.800487916 | 0.381184722 |
| L-Lysine • HCl | 0.799343006 | 0.799343006 | 0.218998084 |
| L-Methionine | 0.20105891 | 0.20105891 | 0.100529455 |
| L-Phenylalanine | 0.399539924 | 0.399539924 | 0.090804528 |
| L-Proline | — | — | 0.173716668 |
| L-Serine | 0.399657436 | 0.399657436 | 0.285469597 |
| L-Threonine | 0.797520467 | 0.797520467 | 0.167899046 |
| L-Tryptophan | 0.078343045 | 0.078343045 | 0.024482201 |
| L-Tyrosine • 2 Na • 2 H_2_O | 0.398192817 | 0.398192817 | 0.111034536 |
| L-Valine | 0.802383249 | 0.802383249 | 0.17071984 |
| Vitamines (mM) | 0.151355461 | 0.151165093 | 0.243337701 |
| Biotin | — | — | 0.000819672 |
| Choline chloride | 0.028571429 | 0.028571429 | 0.021428572 |
| D-Calcium pantothenate | 0.008385744 | 0.008385744 | 0.000524109 |
| Folic Acid | 0.009070295 | 0.009070295 | 0.002267574 |
| Niacinamide | 0.032786883 | 0.032786883 | 0.008196721 |
| Para-Aminobenzoic Acid | — | — | 0.00729927 |
| Pyridoxine hydrochloride | 0.019607844 | 0.019417476 | 0.004854369 |
| Riboflavin | 0.00106383 | 0.00106383 | 0.000531915 |
| Thiamine hydrochloride | 0.011869436 | 0.011869436 | 0.002967359 |
| Vitamin B12 | — | — | 0.00000369 |
| i-Inositol | 0.04 | 0.04 | 0.19444445 |
| Other components (mg·L^-1^) | — | — | — |
| D-Glucose | 1000 | 4500 | 2000 |
| Glutathione | — | — | 1 |
| Phenol Red | 15 | 15 | 5 |
| Sodium pyruvate | 110 | 110 | — |

**Table S5.** Composition of stock solutions used for preparation of amino acid-based dispersants.

| Solution | 1 | 2 | 3 | 4 | 5 |
| --- | --- | --- | --- | --- | --- |
| L-glutamine (mM) | 0 | 4 | 8 | 12 | 20 |
| Sodium bicarbonate (g/L) | 3.7 | 3.7 | 3.7 | 3.7 | 3.7 |

**Table S6.** Instrumental conditions of DLS system for the study of CB-NPs dispersions.

| Continuous DLS | Temperature | 25 ºC |
| --- | --- | --- |
|  | Acquisition time | 3.0 s |
|  | Measurement position | 4.2 mm |
|  | Attenuator | 11 |
| Batch DLS | Temperature | 25 ºC |
|  | Equilibration time | 120 s |
|  | Measurement angle | 173º (NIBS default) |
|  | Acquisition time | 10.0 s |
|  | Position | Automatic seek for optimum conditions |
|  | Attenuator | Automatic seek for optimum conditions |
| Z-Potential | Temperature | 25 ºC |
|  | Model | Smoluchowski (1.50 F(ka)) |
|  | Equilibration time | 120 s |
|  | Acquisition time | Automatic seek for optimum conditions |
|  | Attenuation selection | Automatic seek for optimum conditions |
|  | Voltage | Auto (Máx. 150 V) |

**Table S7.** Optimal instrumental variables and conditions of the AF4 system for the study of CB-NPs dispersions.

| Channel parameters | Length | Long channel (290 mm) | | |
| --- | --- | --- | --- | --- |
|  | Spacer | 350 μm thick | | |
| Membrane | Type | Regenerated cellulose | | |
|  | MWCO | 10 kDa | | |
| Carrier liquid |  | NaN_3_ 0.02 % (w/v) | | |
| Flows | Injection Flow (I_F_) | 0.15 mL·min^-1^ | | |
|  | Tip Flow | 0.50 mL·min^-1^ | | |
|  | Initial Cross Flow (C_F_) | 1.0 mL·min^-1^ | | |
| Times | Focus Time (F_T_) | 1.5 min. | | |
| Injection | Type | Manual | | |
|  | Volume | 18.8 μL | | |
| Detectors |  | DLS and UV-Vis (450 nm) | | |
| Sequence | Mode | Length (min.) | C_F_ (mL·min^-1^) | Gradient |
|  | (1) Injection + Focus | 1.5 | 1.0 | - |
|  | (2) Transition | 1.0 | 1.0 | - |
|  | (3) Elution | 4.5 | 1.0 | - |
|  | (4) Elution | 0.5 | 1.0 to 0.3 | Linear |
|  | (5) Elution | 5.0 | 0.30 to 0.05 | Exp. (0.5) |
|  | (6) Elution | 20 | 0.050 to 0.015 | Linear |
|  | (7) Elution | 2.5 | 0.015 | - |
|  | (8) Elution | 10 | 0 | - |

**Table S8.** AF4 method analytical parameters for studying CB-NPs dispersions with UV-Vis detector at 450 nm.

| Linearity. y = b_1_ x + b_0_ (n = 5) | | |  |  |  |
| --- | --- | --- | --- | --- | --- |
| b_1_ ± s_b1_ | b_0_ ± s_bo_ | R^2^ | Concentration range (μg·mL^-1^) | LOD (μg·mL^-1^) | RSD* (%) (n = 4) |
| 184 ± 5 | 800 ± 500 | 0.998 | 1.3-200 | 0.4 | 3.4 |

* Established at concentration of 25 μg·mL^-1^

**Figure S1.** Relative peak area evolution of the fractograms obtained with UV-Vis detector at 450 nm with ultrasonication time.

**Figure S2.** Fractograms corresponding to a dispersion of CB-NPs N326 in Cel 5 (200 μg·mL^-1^) varying the injection flow (I_F_) and using the DLS detector.

**Figure S3.** Schematic representation of the separation channel of an AF4 system.

**Figure S4.** Fractograms corresponding to a dispersion of CB-NPs N326 in Cel 5 (200 μg·mL^-1^) varying the focus time and using the DLS detector.
